# Comparison of Mitochondrial Mutation Spectra in Ageing Human Colonic Epithelium and Disease: Absence of Evidence for Purifying Selection in Somatic Mitochondrial DNA Point Mutations

**DOI:** 10.1371/journal.pgen.1003082

**Published:** 2012-11-15

**Authors:** Laura C. Greaves, Joanna L. Elson, Marco Nooteboom, John P. Grady, Geoffrey A. Taylor, Robert W. Taylor, John C. Mathers, Thomas B. L. Kirkwood, Doug M. Turnbull

**Affiliations:** 1 Newcastle University Centre for Brain Ageing and Vitality, Institute for Ageing and Health, Newcastle University, Newcastle upon Tyne, United Kingdom; 2 Wellcome Trust Centre for Mitochondrial Research, Institute for Ageing and Health, Newcastle University, Newcastle upon Tyne, United Kingdom; 3 Institute of Genetic Medicine, Newcastle University, Newcastle upon Tyne, United Kingdom; 4 Human Nutrition Research Centre, Institute for Ageing and Health, Campus for Ageing and Vitality, Newcastle Upon Tyne, United Kingdom; 5 Institute for Ageing and Health, Newcastle University, Campus for Ageing and Vitality, Newcastle Upon Tyne, United Kingdom; Max Planck Institute for Biology of Aging, Germany

## Abstract

Human ageing has been predicted to be caused by the accumulation of molecular damage in cells and tissues. Somatic mitochondrial DNA (mtDNA) mutations have been documented in a number of ageing tissues and have been shown to be associated with cellular mitochondrial dysfunction. It is unknown whether there are selective constraints, which have been shown to occur in the germline, on the occurrence and expansion of these mtDNA mutations within individual somatic cells. Here we compared the pattern and spectrum of mutations observed in ageing human colon to those observed in the general population (germline variants) and those associated with primary mtDNA disease. The pathogenicity of the protein encoding mutations was predicted using a computational programme, MutPred, and the scores obtained for the three groups compared. We show that the mutations associated with ageing are randomly distributed throughout the genome, are more frequently non-synonymous or frameshift mutations than the general population, and are significantly more pathogenic than population variants. Mutations associated with primary mtDNA disease were significantly more pathogenic than ageing or population mutations. These data provide little evidence for any selective constraints on the occurrence and expansion of mtDNA mutations in somatic cells of the human colon during human ageing in contrast to germline mutations seen in the general population.

## Author Summary

Mitochondrial DNA encodes essential components of the mitochondrial respiratory chain and is strictly maternally inherited, making it vulnerable to the accumulation of deleterious mutations. To avoid this, mtDNA is subjected to a bottleneck phenomenon whereby only a small number of mtDNA molecules are passed on to the oocyte precursor. These are then amplified to the required number of mtDNA molecules in the mature oocyte, meaning that any mutations may be either lost or rapidly fixed. Purifying selection is thought to be an important protective mechanism against pathogenic mtDNA mutations in the germline, as this is essential for mtDNA stability. It is unknown whether there are any such protective mechanisms in the somatic tissues. To investigate this we have compared the spectrum of mutations present in ageing human colonocytes with those population variants passed through the maternal germline and mtDNA mutations responsible for primary mtDNA disease. We show that pathogenic mtDNA mutations are present at a significantly higher frequency in the somatic cells of the human colon in contrast to variants that have passed though the germline, showing little evidence for purifying selection in the somatic tissues studied here, but strong evidence of this selective mechanism in the germline.

## Introduction

Ageing is a stochastic process commonly defined as the progressive decline in the condition of an organism which is accompanied by a reduction in fertility and an increasing risk of death [1]. Ageing is unlikely to be genetically programmed as evolution theory suggests that nature would not select for a process which is generally harmful to the viability of the organism [2]. The disposable soma theory [3], [4] suggests that there are ‘trade-offs’ between somatic cell maintenance, growth and reproduction. Under constant pressure of natural selection to make optimal use of resources, organisms can afford to make only limited investment in the maintenance and repair of somatic tissues. Metabolic processes associated with tissue maintenance are costly and there is no advantage in keeping somatic cells in good condition beyond the typical survival period in the wild, plus a little reserve. This contrasts with the requirement to protect the germline, where constant high maintenance is needed to maximise capacity for reproduction in the current generation, and to minimise the risk of transferring damage. Nowadays most humans survive long past the life expectancy of our distant ancestors, and experience ageing due to the gradual accumulation of unrepaired damage in somatic cells.

Damage to mitochondrial DNA (mtDNA) resulting in dysfunction of the oxidative phosphorylation (OXPHOS) system has been proposed to be an important contributor to the ageing phenotype [5]. The mitochondrial genome is a circular, double-stranded, ∼16.5 Kb molecule [6] encoding 13 essential polypeptides of the OXPHOS system in addition to 24 mt-RNA genes required for their translation. Its organisation is an example of extreme economy; the vast majority of the molecule is coding and there are no introns, so mutation of the mtDNA is likely to have a functional effect. However, given that there are multiple copies of mtDNA within individual cells, the vast majority of mutations are functionally recessive and a detectable OXPHOS defect occurs only when a critical threshold level of mutant mtDNA is exceeded [7]. A commonly used assay to look for cells or tissues with mtDNA mutations is a histochemical test to demonstrate cytochrome *c* oxidase (COX) activity [8]. Cells lacking functional COX activity commonly contain mtDNA mutations at high levels [9], [10], and whilst this assay may not pick up all mutations it is a good initial screen.

MtDNA is inherited in a non-Mendelian manner exclusively through the female line [11] and bi-parental recombination, if it has ever occurred, has left no footprint on the phylogeny [12]. Asexual inheritance should leave the mitochondrial genome vulnerable to extinction due to the irreversible accumulation of mtDNA mutations, a process known as Muller's Ratchet [13]. In the absence of recombination, the only means to prevent this outcome is a selection process against mutants which is strong enough to substantially abate the relatively high mutation rate experienced by mtDNA. In principle, selection against mtDNA mutations can act at several levels since cells typically contain large numbers of mtDNA molecules which have a replication cycle that is not strictly coupled with that of the cell itself [14]. Selection against mtDNA mutations could therefore arise (i) through direct competition between mtDNA variants within the cell, (ii) through selection at the cell level, if mutants affect cellular survival and/or division, or (iii) through selection at the whole organism level, via effects on fitness of the individual.

A distinctive mechanism that is thought to enhance the power of selection to keep mtDNA mutations in check within the germline is the mitochondrial “bottleneck” [15]. The bottleneck event effectively samples mtDNA molecules ensuring only a small proportion are selected to form the mtDNA pool [16] in the mature oocyte which is passed to the offspring. The mammalian oocyte contains over 100,000 mtDNA molecules which do not replicate during early embryogenesis [11]. They segregate during cell division events and when the primordial germ cells are formed they contain only 200–400 mtDNA molecules [17]. As primordial germ cells differentiate into mature oocytes which will transmit their mtDNA to the next generation [18], there is re-initiation of mtDNA replication of a small subset of mtDNA molecules [19]. Currently it is a matter of intense debate as to whether this expansion occurs during embryogenesis or post-natal folliculargenesis [17], [19], but regardless of the exact timing, deleterious mutations will either be lost or they will be more strongly exposed to natural selection and, if not eliminated, become fixed more rapidly [20]. Germline selection adds to the purifying selection of mtDNA that had already been shown to occur in human populations by comparison of different lineages [21], [22]. Together the population studies and experimental studies in the mouse [23], [24] demonstrate a multi-level selective process which can act to remove deleterious and slightly deleterious variants.

While the full repertoire of mechanisms to combat mtDNA mutation accumulation act within the germline, there is less of an imperative to prevent such accumulation within the soma, and there are fewer opportunities to do so. Somatic mtDNA mutations have been shown to accumulate to high levels in a number of human ageing tissues including brain, heart, skeletal muscle, colon, stomach and liver, leading to OXPHOS dysfunction [10], [25]–[29]. Thus, the accumulation of mtDNA mutations in the soma of ageing humans may contribute to the decline in cellular functionality commonly seen during ageing. In addition mouse models with increased levels of mitochondrial DNA (mtDNA) mutations [30]–[32] show a premature ageing phenotype suggesting that mitochondrial defects are important in the ageing process.

Although the accumulation of age-associated somatic mtDNA mutations is not subject to the full set of selective constraints that operate in the germline, it is possible that selection at the intracellular and intercellular levels could influence the spectrum of mutations that is seen. Experimental support for this hypothesis is limited and information on the pattern of mtDNA mutations which occur with age is likely to inform our understanding of the underlying mechanisms that might be involved. Previous studies have shown that there are differences in the type of mutations detected with ageing in post-mitotic and mitotic tissues. In post-mitotic tissues the predominant mutated species are large-scale mtDNA deletions [33]–[36], whereas in dividing cells mtDNA point mutations are commonly identified [9], [26], [27], [37]–[39]. As large-scale mtDNA deletions are rarely transmitted through the germline we investigated possible selective constraints only on mtDNA point mutations which occur somatically during human ageing by analysing location, mutation type and predicted mutation pathogenicity using the human colon as a model ageing system. We have previously generated a number of mtDNA sequences from ageing colonocytes [9], [10], [38] providing an excellent data set of age-related somatic mutations for investigation. We then compared the mutational spectra with germline mtDNA point mutations seen in a geographically-comparable human population and well-characterised pathogenic mtDNA point mutations which are documented causes of primary mtDNA disease phenotypes.

## Results

### A dataset of age-related mtDNA mutations

In order to ensure that we had a high quality data set of age-related somatic mtDNA mutations to use in the subsequent analyses, 72 individual cells from 9 participants over 70 years of age were sequenced, and the presence of clonally-expanded mtDNA mutations was documented. These data were combined with those which we have published previously on human colon (age range 70–76 years) [9], [10], [38]. In our previous studies cells were sequenced based on the absence or presence of COX activity. The majority of cells were COX deficient as these studies aimed to detect the underlying genetic defect in these cells. This strategy increases the likelihood of detecting pathogenic mtDNA mutations. Here we wanted to obtain a more complete picture of the mutational spectrum across the human colon including non-pathogenic mutations and those which do not affect COX activity, therefore we sequenced an additional 48 COX positive cells and 24 COX deficient cells. This gave a total of 156 whole mitochondrial mtDNA sequences (71 sequences are from COX positive cells and 85 from COX deficient cells [26], [32], [33]). These sequences yielded a collection of 129 clonally-expanded mtDNA point mutations which were found in 89 different cells. 67 of the cells in this dataset did not contain any clonally expanded mtDNA mutations. Heteroplasmy levels were estimated based on the ratio of peak heights on the electropherograms. A relatively high proportion of the mutations (39%) were present at homoplasmic levels, however we believe that this is due to the fact that crypts were selected based on their COX activity rather than any selective mechanism. COX deficient cells contained 88% of the homoplasmic mutations. This highlights the fact that the majority of mtDNA mutations require a high level of mutation to be reached before a biochemical defect is observed [7]. Of the cells which contained mtDNA mutations, the majority (61) contained only one mutation, 20 contained 2 mutations, 5 contained 3 mutations and 3 contained 4 or more mutations. Where there were multiple mutations present only 5 cells contained mutations that were present at the same levels of heteroplasmy and in all cases this was 100%, the remaining cells had varying levels of heteroplasmy, indicating that each mutation occurred at different times or on different molecules. All mutations in the data set are listed in Table S1 along with their COX status. Only mutations in the coding region were included in the following analyses, as mutations in these regions are more likely to have deleterious effects. From here on the somatic mutations detected in the human colon will be referred to as ‘somatic’ or ‘age-related’ mutations.

### Age-related mtDNA mutations occur randomly within the genome

To investigate the location of the changes on the mtDNA of age-associated mutations they were grouped by base pair number (per 2000 bases; 1–2000, 2001–4000 etc). Based on the assumption that all groups had an equal chance of containing a mutation, chi-squared analysis showed no significant difference in the observed and expected frequencies (p = 0.293) which supports the conclusion that age-associated mutations are located randomly throughout the mitochondrial genome.

**Figure 1:**
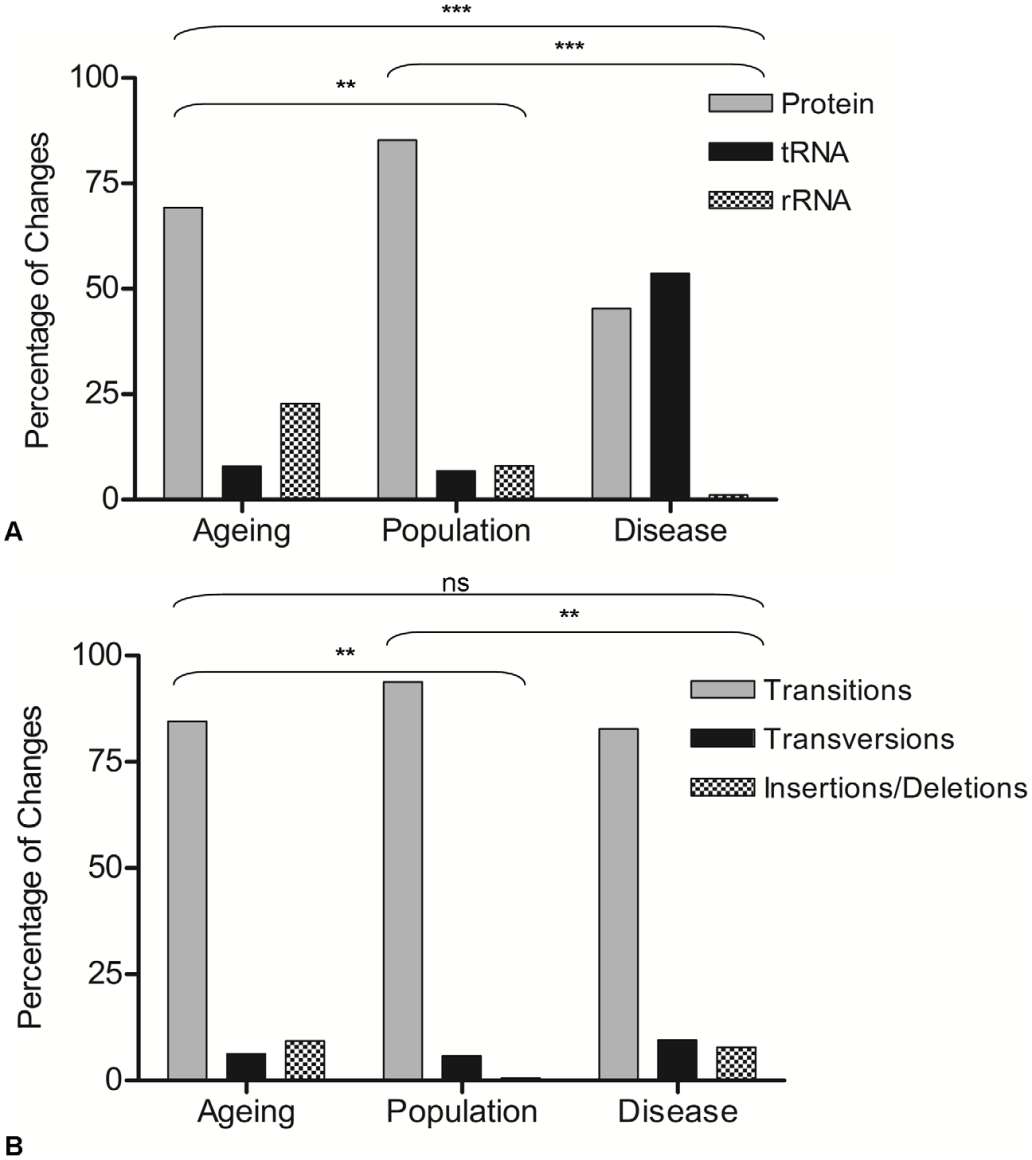
Gene location and types of mutations observed in ageing, population, and disease. A: Gene location of mutations. Data are represented as the percentage of the total coding region mutations. Contingency analysis with Bonferroni correction for multiple testing was carried out on the frequencies of the changes in each gene type (ageing n = 117, population n = 182, disease n = 176). Thresholds for statistical significance are; ***<0.0003, ** 0.003, * = 0.017. B: Types of changes observed in ageing, population and disease. Data are represented as the percentage of the total coding region mutations. Contingency analysis with Bonferroni correction for multiple testing was carried out on the frequencies of the changes in each mutational category. Thresholds for statistical significance are; ***<0.0003, ** 0.003, * = 0.017.

To be confident that these mutations truly were random they were then grouped according to gene status (e.g. whether they occurred within mt-tRNA, mt-rRNA or protein encoding genes). Similarly, the difference between the observed and expected frequencies was not significant (p = 0.129) (chi-squared analysis comparing observed to expected frequencies based on the proportion of the genome occupied by each gene type). A comparison was then made with the disease (full mutation list in Table S2) and population variants (full list in Table S3) (Figure 1A). In the disease-associated mutation group, there was a significantly higher proportion of mutations in mt-tRNA genes (53%) compared to other gene types (p<0.0003). This has been reported previously in other studies [23], [40]. In the rare population variants, the majority of mutations were in the protein encoding genes (85%) (p<0.003), probably because a high proportion of the changes in the protein encoding genes of the population predict synonymous amino acid changes which are non-pathogenic and are therefore unlikely to be selected against in the germline. The nature of changes (e.g. transitions, transversions and insertions/deletions) was then examined in the three groups. In all cases, the overwhelming majority of changes were transitions (90%, 94% and 87% in age-related mutations, population variants and disease mutations respectively). There was a significantly higher frequency of insertion and deletion mutations in the both the age-related mutation group compared to the population variants, and the disease mutation group compared to the population variants (p<0.003 in both cases, contingency analysis with Bonferroni correction for multiple testing, Figure 1B).

### Non-synonymous mtDNA mutations are present more frequently during ageing and mtDNA disease than in populations

**Figure 2:**
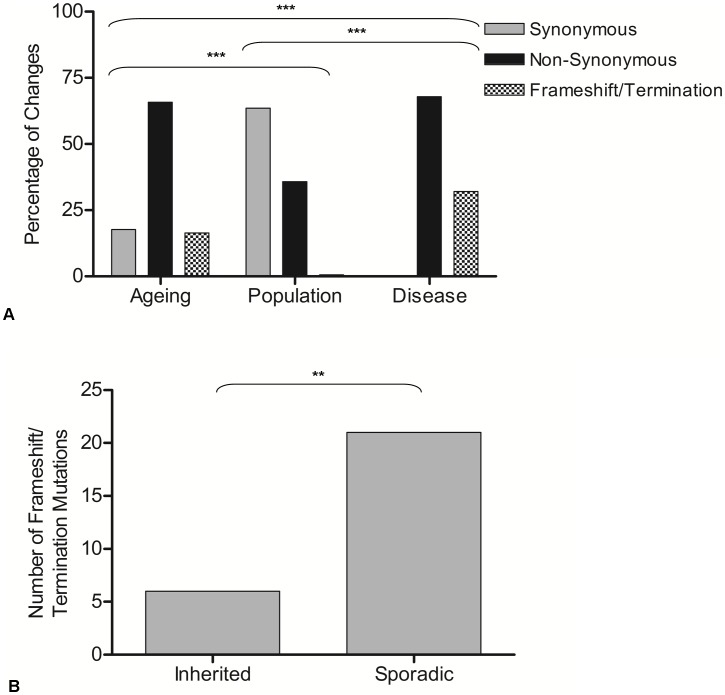
Genetic consequences of mutations observed in ageing, population, and disease. A: The percentage of changes which predict synonymous, non-synonymous and premature termination codons or frameshifts in protein encoding genes. Contingency analysis with Bonferroni correction for multiple testing was carried out on the frequencies of the changes in each gene type (ageing n = 81, population n = 155, disease n = 76). Thresholds for statistical significance are; ***<0.0003, ** 0.003, * = 0.017. B: Frequency of termination codon and frameshift mutations in inherited and sporadic disease-causing mtDNA mutations. Chi-squared analysis showed a significantly higher frequency such mutations in sporadic than inherited cases (p = 0.003).

Purifying selection models have shown that, in the germline, selection is strongest against mutations in protein-encoding genes which cause an amino acid change [23], those which predict a frameshift, or those which cause premature translation termination [19]. A comparison of such mutations in the three data sets was made (Figure 2A). This revealed a significantly higher number of non-synonymous (65.4%) and frameshift/premature termination codons (16.5%) in the ageing cells compared with the rare population variant group (34% and 0.6% respectively) (contingency analysis with Bonferroni correction for multiple testing, p<0.0003). This suggests that human somatic cells are not protected from accumulation of age-related deleterious mutations in the same way as has been shown during germline transmission of mtDNA mutations in the mouse [23]. Comparison of age-associated mutations and disease-causing mutations revealed a significantly higher number of frameshift/premature termination codons in the disease group (32%) (p<0.0003).

To investigate further the apparent selection-free environment, from the stand point of mtDNA mutations, in somatic cells, protein encoding disease-causing mutations were grouped according to whether these had been reported as ‘inherited’ or ‘sporadic’ mtDNA mutations (as defined in the original reports, see Table S2 for details). Whilst inherited mtDNA mutations are passed through the germline, it is proposed that sporadic mutations occur during early embryological development beyond the point of the genetic bottleneck [17], [19]. Further examination of frameshift and termination codon mutations (which might be predicted to be the most pathogenic) showed that a significantly higher number were sporadic (78%) rather than inherited (22%) (chi-squared analysis, p = 0.003) (Figure 2B), supporting the idea that these arose after the selective check point.

### Age-related and disease-causing mutations in protein-encoding genes are more pathogenic than rare population variants

**Figure 3:**
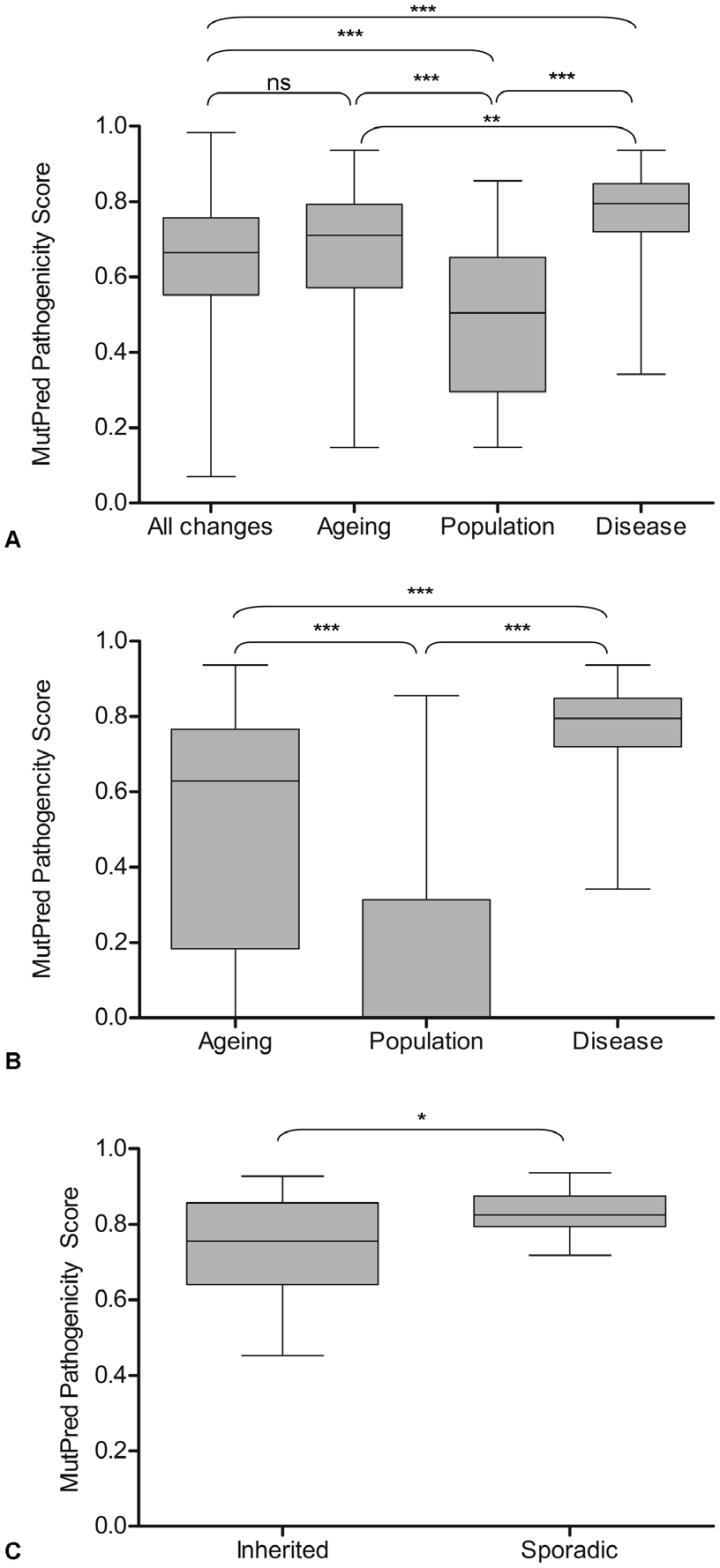
MutPred pathogenicity scores of mtDNA mutations in ageing, populations, disease, and all possible mtDNA mutations. A: The distribution of pathogenicity scores in ageing, populations, disease and all possible mutations. Results of Wilcoxon rank sum analysis are shown. With six tests, the threshold for significance is 0.05/6 = 0.008, ** = p<0.0017, *** = p<0.00017. B: The distribution of pathogenicity scores including synonymous mtDNA mutations which were assigned a pathogenicity score of 0, in ageing, population and disease. Results of Wilcoxon rank sum analysis are shown. With three tests the threshold for significance is 0.05/3 = 0.017, ** = p<0.003, *** = p<0.0003. C: The distribution of pathogenicity scores in sporadic and inherited disease-causing mtDNA mutations. Results of Wilcoxon rank sum test are shown,* p<0.05.

Pathogenicity of the non-synonymous mutations in the three groups was predicted using MutPred software [41], and the groups compared. The MutPred score is determined by a set of features reflecting protein structure, evolutionary conservation, presence of functional residues and amino acid biases at the substitution site and its neighbours. Amino acid variants are identified by comparison to their counterparts defined by the rCRS sequence [42]. Mutations are assigned a score between 0 and 1, with higher pathogenicity scores corresponding to a greater likelihood that the amino acid change is pathogenic. Only non-synonymous protein encoding gene mutations were used in this analysis because the software is unable to analyse the pathogenicity of frameshift mutations and those which predict a premature termination codon. In addition, pathogenicity scores for all possible amino acid changes in the mitochondrial genome, predicted by the MutPred software (published as a supplementary table in [43]) were included in these analyses (Figure 3A). There was no significant difference in the mean pathogenicity score between the age-related mutations (0.651±0.203 (sd), n = 52) and all possible mutations (0.642±0.154, n = 24206) (p = 0.94, Wilcoxon rank sum test). This evidence supports the hypothesis that there are no selective constraints on mutations which accumulate in the human colon during ageing. The age-related mtDNA mutations had significantly higher MutPred scores (0.651±0.203, n = 52) than the rare population mtDNA mutations (0.481±0.202, n = 53) (p<0.00017) but significantly lower scores than disease-associated mtDNA mutations (0.761±0.13, n = 54) (p = 0.0016), supporting the hypothesis that these mutations occur at random during the life-course and their expansion is not curtailed by purifying selection.

To obtain a more complete picture of the pathogenicity of the protein encoding mtDNA mutations, the synonymous mutations in the ageing and population groups (there were none in the disease group) were assigned a pathogenicity score of 0 (as they do not change an amino acid and are therefore very unlikely to have a functional effect) and included in the analysis (Figure 3B). There were significant differences between the three groups (Wilcoxon rank sum tests, p<0.0003 in all cases) with pathogenicity scores highest in the disease-associated mutation group (0.761±0.13, n = 54) followed by age-associated mutations (0.513±0.323, n = 66) and population variants (0.171±0.26, n = 149). Given the very high pathogenicity scores predicted in the disease mutation group, mutations in this group were separated into inherited and sporadic mutations and the mean scores compared (Figure 3C). Mean MutPred pathogenicity score was significantly higher in the sporadic mutation group (0.831±0.059, n = 16) compared with the inherited mutations (0.737±0.136, n = 26) (p = 0.02, Wilcoxon rank sum test).

### MtDNA mutations in other somatic tissues

All of the above analyses have been based on somatic mtDNA point mutations detected in individual ageing human colonic crypts as this is the most comprehensive dataset of such mutations to date. However it is known that there are differences in mutation occurrence and accumulation between different tissues. For example clonally expanded point mutations and not large scale deletions are commonly found in dividing tissues [10], whereas large-scale deletions are the predominant species of mtDNA mutation in post-mitotic tissues [33], [44]. In addition, it has been shown that in mice with two different mitochondrial genotypes, there is tissue specific accumulation of one genotype over another [45]. Therefore a literature search was carried out and a dataset of mtDNA point mutations in normal ageing tissues compiled. Mutations were only included if they had been detected using the same methodology and quality control analyses as the mutations detected in the colonic epithelium, and were in normal, not tumour tissues. A total of 24 mutations were included from stomach epithelium [27], prostatic epithelium [46], bladder urothelium [47], small intestinal epithelium [48], pancreas [49] and liver [26], [37]. All mutations were detected in COX deficient cells. Full details of all mutations are in Table S4. The mutations detected had a similar mutation spectrum as the colon mutations, with 92% of the mutations being transitions, 4% transversions and 4% single base pair deletions. Due to the small size of the dataset, it was not possible to do a detailed comparison of the location of the mutations, but mutations were found in all gene types (protein encoding, mt-rRNA and mt-tRNA genes). There were 8 non-synonymous protein encoding mutations, all in COX encoding genes, which had a mean MutPred score of 0.811±0.03. This was then compared to the MutPred score for COX-encoding gene mutations in COX deficient cells of the colon. To exclude the effects of multiple mtDNA mutations within a cell, mutations were only included if they were either the only mutation in the cell, or the only coding-region mutation in the cell. There was no significant difference in the MutPred score between the mtDNA mutations in the colonic crypts (0.743±0.03, n = 10) and those in the other tissues analysed (p = 0.07, Wilcoxon rank sum test). These data provide evidence that occurrence and clonal expansion of pathogenic protein encoding mutations with age is not restricted to only the colonic epithelium, but is likely to be relevant to other mitotic tissues.

## Discussion

The aim of this study was to compare the selective constraints on the occurrence and expansion of age-related mtDNA mutations in the human colon with those in the general population and those which cause primary mtDNA disease. Purifying selection in the germline has been demonstrated in two studies using two different mouse models. One group utilised a mouse with a homozygous knock-in allele expressing a proof-reading- deficient mtDNA polymerase γ which, as a consequence, accumulates high levels of random mtDNA point mutations [23]. This mouse was backcrossed with mice with wild-type mtDNA polymerase and the offspring showed rapid elimination from the germline of non-synonymous protein encoding mutations. A complementary study generated a mouse model of mtDNA disease with 2 mtDNA mutations, one relatively mild mtDNA mutation and one severe mtDNA mutation. Only the mild mutation was transmitted through the germline to the next generation. [24]. The reduction of mtDNA copy number at the mtDNA bottleneck followed by huge expansion of a small number of mtDNA molecules allows rapid fixation of new mtDNA mutations and the opportunity to select against deleterious mutants by unknown mechanisms which could operate at the DNA, organelle or cellular level (primordial germ cell lineage) [50].

Our study has shown no evidence for the action of purifying selection on age-related mtDNA mutations in a number of different tissue types, which we observed are distributed randomly throughout the mitochondrial genome. Further, frequency of age-related mtDNA mutations in each gene is equal to the proportion of the mitochondrial genome occupied by that gene suggesting that there is no functional selection. There was a predominance of transition mutations compared with transversions and insertions/deletions, with 65% of those transitions being C∶G to T∶A mutations. These mutation types are likely to be due to errors during replication of mtDNA and/or spontaneous cytosine deamination [51], the remaining transitions are likely to be due to replication errors. There was only one C∶G to A∶T mutation in the dataset, suggesting that there is little contribution of 8-oxo-deoxyguanine mediated mutagenesis, thus casting doubt on the importance of oxidative damage in ageing, at least in this tissue type. The mutational spectrum here probably reflects the initial mechanism of mutation occurrence rather than selection during clonal expansion.

There were differences between the somatic age-related mtDNA mutations and the mutations we observe in the general population. The most prominent differences are the expansion of non-synonymous protein encoding mutations, including frameshift and termination mutations, in somatic cells compared with the germline, and the higher pathogenicity scores of the age-related mutations. These observations provide evidence that there is little or no selection against pathogenic mutations in the soma, whereas the germline is relatively protected from such mutations by the mtDNA bottleneck [52]. Pathogenic mtDNA mutations in both mt-tRNA genes and mt-mRNA genes which cause disease can either be maternally- inherited or sporadic. Our predicted pathogenicity analysis of the mutations in the protein encoding genes shows that those which are sporadic, and therefore occur post-bottleneck, are more pathogenic than those which are inherited. The high prevalence of mt-tRNA mutations which cause primary mtDNA disease suggests that they are passed through the germline more easily than mt-mRNA variants, perhaps because in the majority of cases they produce a less severe phenotype [7], [40]. The mouse study by Stewart et al., also showed no evidence of purifying selection on mt-tRNA mutations [23].

A number of the mtDNA mutations detected in the human colon were also associated with loss of COX activity, and COX deficiency is a common observation in patients with mtDNA disease. This suggests that mitochondrial quality control systems such as mitophagy have limited efficiency to remove dysfunctional mitochondria to restore normal function. This is in contrast to a study in which Parkin, an E3 ligase which selectively translocates to depolarised mitochondria and activates their elimination by autophagy, was continually overexpressed in a cybrid cell line containing a mutation in *MT-CO1* [53]. Parkin overexpression resulted in selective degradation of dysfunctional mitochondria, an increase in the proportion of wild-type mtDNA, and restoration of COX activity. In our study we see accumulation of pathogenic mtDNA mutations in individual cells. The difference could either be because mtDNA mutations which lower membrane potential to the extent by which Parkin becomes activated are selected against and are therefore not able to accumulate in individual somatic cells, or that physiologically normal levels of Parkin do not have the same effect as Parkin over expression.

These studies have shown that mutations in genes other than *MT-CO* genes can result in COX deficiency. Disease-associated mutations in mt-tRNA genes are commonly associated with COX defects [54]and mutations in mt-rRNA genes could be causing a translational defect to explain the COX deficiency. We have previously shown that multiple respiratory chain complex deficiency is a common occurrence in the human colon, with approximately 50% of crypts exhibiting COX deficiency also showing a loss of complex I and III subunits immunohistochemically [38]. This multiple respiratory chain complex deficiency could therefore be attributed to mutations in any of the mt-mRNA genes, explaining why we observe COX deficiency associated with mutations in non-*MTCO* genes. Multiple biochemical defects have been observed in patients with mutations in structural subunits of complex I [55] and complex III [56], [57] highlighting the fact that complexes of the respiratory chain do not simply exist as simple entities but associate to form ‘supercomplexes’ [58], [59]. Moreover, it has recently been shown that if one component complex is absent, supercomplexes are not formed [58] and that the incorporation of a defective structural component, such as a truncated form of COX1, can lead to the rapid degradation of supercomplexes and downregulation of other mitochondrially-encoded respiratory chain subunits [60]

From an evolutionary perspective, our data are consistent with the predictions of the disposable soma theory of ageing [3]. By comparing the somatic age-related mtDNA mutations which clonally expand within individual cells of the colon with those passed through the germline in populations we have shown considerable evidence supporting the hypothesis that there is selection against pathogenic mtDNA mutations in the human germ-line but little evidence of any such selection in the somatic tissues studied here.

As there are multiple copies of the mitochondrial genome in an individual cell, any mutation occurring in one molecule will be functionally recessive [61]. A functional defect becomes apparent only when a mutation expands clonally to high levels in a cell (typically >70%) [7]. Numerous studies in human tissues have shown an age-dependent increase in the fraction of individual cells which are respiratory-chain deficient [10], [25], [27]–[29], [49] and that the cause of respiratory chain deficiency is clonally expanded somatic mtDNA mutations [10], [27], [62]. Since our data suggest that there is no obvious imprint of selection on the spectrum of age-related mtDNA mutations in the human colon, it remains of interest to consider how such clonal expansion occurs. One leading hypothesis, supported by computational models, is that clonal expansion occurs simply through a process of random genetic drift [63]. Unlike nuclear DNA, mitochondrial DNA is replicated independently of the cell cycle and not all molecules are always replicated (relaxed replication) [14]. The random genetic drift hypothesis suggests that relaxed replication of mtDNA coupled with random degradation of some mtDNA molecules can lead to one mutant genotype becoming the dominant genotype of the cell. A study using heteroplasmic mice containing two different mtDNA genotypes measured the relative contribution of each genotype in individual colonic crypts over time. The study showed that in individual crypts from 4 month old mice there was a mixture of the two genotypes, however in the crypts of the 15 month old mice two distinct crypt populations were observed with one or other of the genotypes predominating. This experimental data fits with a model of neutral drift [45]. In humans, the random genetic drift model predicts that clonal expansion takes decades to occur [63]. Experimental data from a number of tissues has shown that respiratory chain deficient cells are not detectable in humans before approximately age 35 years [10], [28], and in the human colon we have shown previously that such defects are present in one third or more of colonocytes from those aged 70+ [10]. This, coupled with the mutational spectrum detected in the human colon is supportive of the ‘early mutation, slow expansion’ theory of mitochondrial ageing [64].

In conclusion, these investigations have shown significant differences in the spectrum and predicted pathogenicity of mtDNA mutations in the somatic tissues studied here which are associated with ageing, rare population variants which have passed through the germ-line and those pathogenic mtDNA mutations associated with disease. We found little evidence for purifying selection in the somatic tissues investigated, whereas there is strong evidence for purifying selection in the germ-line.

## Materials and Methods

### Generation of a dataset of age-associated somatic mtDNA mutations

Colorectal mucosal samples were collected from the same anatomical site (10 cm from the anal verge) from subjects (n = 9, age range 71–78 years) undergoing colonoscopy for disturbed bowel function in whom no evidence of bowel disease was identified (BORICC 1 Study). Informed consent was given by all participants in the study and ethical approval was obtained from the Northumbria NHS Trust Local Research Ethics Committee (project reference 04/Q0902/6/).

Single colonocytes were laser-microdissected into sterile 0.5 ml PCR tubes using a Leica Laser Microdissection (AS-LMD) System. Following centrifugation (7000×g for 10 min), the cell was lysed in 14 µl of cell lysis buffer (50 mM Tris-HCl pH 8.5, 1 mM EDTA, 0.5% Tween-20, 200 ng/ml proteinase K) at 55°C for 2 hours and then 95°C for 10 minutes to denature the proteinase K. The entire sequence of the mitochondrial genome from microdissected colonocytes was determined using the single cell lysate as the DNA template. Two rounds of PCR were carried out as previously described [10]. PCR products were cycle sequenced using ABI BigDye chemistries per standard manufacturer's protocols and analysed on an ABI3100 genetic analyser (Applied Biosystems). Sequences obtained were compared with the rCRS [42] and the homogenate sequence for each patient (phylogenetic quality control), using SeqScape software (Applied Biosystems). Due to potential problems with low mtDNA copy number PCR [65], all putative mutations were re-amplified from the original DNA lysate and were sequenced in both the forward and reverse directions. This strategy aimed to eliminate any errors due to either amplification of nuclear pseudogenes or errors introduced by the DNA polymerase during PCR amplification. These mutations were added to an existing database of published somatic mtDNA mutations detected in the ageing colon [9], [10], [38]. A full list of mutations can be found in Table S1.

### Generation of a disease-associated mtDNA mutation data set

Due to the inherent difficulty in assigning pathogenicity to reported mtDNA disease-associated mutations we applied strict criteria when assembling a database of such mutations for the purpose of this study. Protein encoding mutations were included only if they were assigned as ‘probably’ or ‘definitely pathogenic’ using a validated scoring system designed in this laboratory [66]. Our database includes all such mutations previously assigned pathogenicity scores by Wong in 2007 [67], as well as 4 more recently published mutations which were shown to score in these categories [68]–[71]. We also included tRNA mutations proven to be pathogenic by a similar scoring system [72]. Only two rRNA mutations have been confirmed on mitomap to be pathogenic and therefore only these two were included in the analysis [73]. A full list of mutations can be found in Table S2.

### Rare population mtDNA variant dataset

All of the participants whose data were included in the ageing mutation database were of European haplotype therefore the 100 European coding region sequences were chosen. The 100 European sequences were randomly selected reflecting the European haplogroup distribution. The sequences were drawn from the MitoKor database, the changes selected as the rare population variants (Table S3) had been classified as “private” for the purposes of the MitoKor database. In the context of the “Mitokor” database a private change was one that was seen in a single individual only from a continental group i.e. for the European sequences it was seen in only one from 433 individuals. In the time since the generation of the MitoKor database there has been a vast increase in the amount of available sequences but the variants analysed remain a pool of rare variants on European haplogroup backgrounds. In addition, all sequencing to yield data for the Mitokor database was carried out with fourfold redundancy which provides confidence that these are rare variants not sequencing errors.

### Pathogenicity scoring

The pathogenicity of non-synonymous mutations in the 13 protein encoding genes was predicted using the computational software MutPred [41], which is based on the established SIFT method [74], [75]. The MutPred scores for all 24,206 possible amino acid variations published by Pereira et al [43] were also included in these analyses.

## Supporting Information

Table S1 Somatic mitochondrial DNA point mutations detected in ageing human colonic crypt cells.
(XLSX)

Table S2 Mitochondrial DNA point mutations which are reported to cause human mitochondrial disease.
(XLSX)

Table S3 Rare population variants in 100 European coding region mitochondrial DNA sequences.
(XLSX)

Table S4 Somatic mitochondrial DNA point mutations reported in ageing human tissues.
(XLSX)
